# False Lumen Flow Patterns and their Relation with Morphological and Biomechanical Characteristics of Chronic Aortic Dissections. Computational Model Compared with Magnetic Resonance Imaging Measurements

**DOI:** 10.1371/journal.pone.0170888

**Published:** 2017-01-26

**Authors:** Paula A. Rudenick, Patrick Segers, Victor Pineda, Hug Cuellar, David García-Dorado, Arturo Evangelista, Bart H. Bijnens

**Affiliations:** 1 Physense, Universitat Pompeu Fabra, Barcelona, Spain; 2 University Hospital and Research Institute Vall d’Hebron, Universitat Autònoma de Barcelona, Barcelona, Spain; 3 Biofluid, Tissue and Solid Mechanics for Medical Applications, Institute Biomedical Technology, Ghent University, Ghent, Belgium; 4 ICREA, Barcelona, Spain; University of California San Diego, UNITED STATES

## Abstract

Aortic wall stiffness, tear size and location and the presence of abdominal side branches arising from the false lumen (FL) are key properties potentially involved in FL enlargement in chronic aortic dissections (ADs). We hypothesize that temporal variations on FL flow patterns, as measured in a cross-section by phase-contrast magnetic resonance imaging (PC-MRI), could be used to infer integrated information on these features. In 33 patients with chronic descending AD, instantaneous flow profiles were quantified in the FL at diaphragm level by PC-MRI. We used a lumped-parameter model to assess the changes in flow profiles induced by wall stiffness, tear size/location, and the presence of abdominal side branches arising from the FL. Four characteristic FL flow patterns were identified in 31/33 patients (94%) based on the direction of flow in systole and diastole: *B*_*A*_ = systolic biphasic flow and primarily diastolic antegrade flow (n = 6); *B*_*R*_ = systolic biphasic flow and primarily diastolic retrograde flow (n = 14); *M*_*A*_ = systolic monophasic flow and primarily diastolic antegrade flow (n = 9); *M*_*R*_ = systolic monophasic flow and primarily diastolic retrograde flow (n = 2). In the computational model, the temporal variation of flow directions within the FL was highly dependent on the position of assessment along the aorta. FL flow patterns (especially at the level of the diaphragm) showed their characteristic patterns due to variations in the cumulative size and the spatial distribution of the communicating tears, and the incidence of visceral side branches originating from the FL. Changes in wall stiffness did not change the temporal variation of the flows whereas it importantly determined intraluminal pressures. FL flow patterns implicitly codify morphological information on key determinants of aortic expansion in ADs. This data might be taken into consideration in the imaging protocol to define the predictive value of FL flows.

## Introduction

Aortic dissection (AD) is a life-threatening condition. Whereas medical treatment might be a better choice than surgery in patients with acute type B ADs without complications, up to 50% of these patients show progressive false lumen (FL) enlargement and spontaneous rupture during the chronic stage [1].

On the one hand, modifiable morphological parameters, such as a large interluminal communicative area [2,3] and incidence of visceral arteries arising from the FL [4]; and non-modifiable biomechanical features, such as aortic wall stiffness [4]; have been suggested as predictor of progressive aortic dilatation. However, many of these parameters are difficult to measure with the limitation of the available imaging techniques plus different techniques provide inconsistencies between measurements. On the other hand, given that FL patency seems to be a main factor in late complications in chronic ADs [5,6] and that aortic dilatation has been linked to FL pressures and flows [5,7–9], there is increasing clinical interest in looking at the predictive value of FL flow patterns.

**Fig 1:**
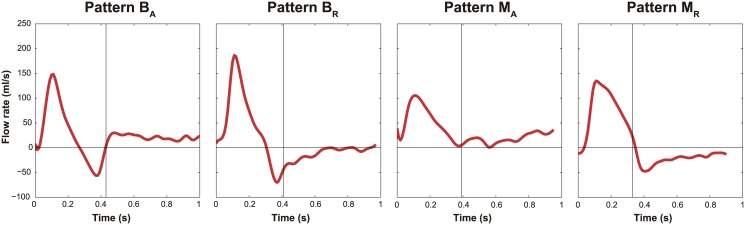
Typical false lumen flow patterns. Four representative false lumen flow patterns (assessed at the diaphragm) observed in our patients in follow-up. They can be classified based on the direction of flow in systole and diastole; *B*_*A*_: systolic biphasic flow and primarily diastolic antegrade flow; *B*_*R*_: systolic biphasic flow and primarily diastolic retrograde flow; *M*_*A*_: systolic monophasic flow and primarily diastolic antegrade flow; *M*_*R*_: systolic monophasic flow and primarily diastolic retrograde flow. Antegrade flows are positive and retrograde flows are negative.

Phase-contrast magnetic resonance imaging (PC-MRI) provides quantitative and qualitative intraluminal flow information in AD. Several studies [10–12], as well as our own clinical observations (Fig 1), revealed significant FL flow pattern differences among individual patients, where complex patterns and the amount of volume in the FL, have been associated to aortic expansion [5,9,11]. Therefore, FL flow might have a predictive role in the long-term evolution. However, many studies are merely descriptive, technically limited, and not prospective, and even when they tend to relate the flow characteristics in patients with the rate of aortic expansion, they lack in linking and integrating them to the underlying morphological and biomechanical information. Therefore, little remains known on the determinants and relevance of these flow pattern differences in chronic ADs as well as how these can/should be studied with imaging.

We hypothesized that FL flow patterns can be used to infer integrated information on morphological/biomechanical configuration in ADs, so that typical flow variations observed in most patients depend on biophysical/morphologic parameters known to be key for assessing aneurysmal dilatation in ADs: wall stiffness [4,13], occurrence of abdominal side branches originating from the FL [5], tear size and tear location [2].

For this, we evaluated a patient population with chronic AD by PC-MRI and used a lumped-parameter computational model to determine the individual contribution of each parameter in the absence of measurement variability and other sources of noise. Although computational fluid dynamics (CFD) models, with rigid wall assumptions, are extensively used in the study of hemodynamics in ADs, a lumped-parameter model is a good model of choice for the required level of detail of this study, which aims to explain observations from PC-MRI in patients, where only quantification of average flow characteristics is done at some planes along the dissection.

We identify the characteristic FL flow patterns in a population of chronic descending ADs as well as which are the key factors that potentially determine them, thus aiming to explain observations from PC-MRI in a typical population in descending ADs. This might provide suggestions for optimal assessment and interpretation of flow patterns in clinical practice and a new means to relate observed alterations to their determining factors and association with outcome.

## Materials and Methods

### Patients and MRI protocol

The study population consisted of 33 consecutive patients with chronic dissection of the descending aorta during long-term follow-up in our hospital: 24 surgically-treated type A dissections and 9 medically-managed type B dissections. The study was approved by the University Hospital Vall d’Hebron institutional review committee and patients gave their written informed consent.

The MRI studies were performed 3–12 months after the acute phase on a 3T scanner (Trio, Siemens, Germany) equipped with fast gradient system characterized by a peak gradient amplitude of 45 mT/m and a maximal slew rate of 200 T/m/s. For flow quantification, a breathhold segmented phase-contrast gradient-recalled echo through-plane sequence, with a temporal resolution of 25–35 ms and retrospective gating, was performed (TR = 57.6 ms; TE = 1.9 ms; flip angle = 30 degrees; parallel imaging with an acceleration factor of 2; matrix size = 192 mm^2^; field of view = 330 mm^2^; voxel size = 1.7x1.7x5 mm^3^; slice thickness = 5 mm; bandwidth = 550 Hz/pixel; 24–30 frames per cardiac cycle depending on heart rate; velocity encoding = 130 to 200 cm/s). Flow velocity-encoded MRI was performed in an oblique transverse plane perpendicular to the course of the descending aorta at the level of the diaphragm. This location was chosen because minimal flow disturbance due to proximal tear flow or major branches in the vicinity was expected.

Flow velocity-encoded MRI data were analyzed and regions of interest in both the TL and the FL were manually segmented (Argus flow software, Siemens, Germany). Since the mean pixel value in both regions was proportional to the flow velocity, the instantaneous flow rate for each frame was computed as the product of the cross-sectional area and the average velocity. We consequently obtained a flow rate–time curve from the summation of the instantaneous flow rates for all the frames through the entire cardiac cycle.

### Representative aortic geometry

**Fig 2:**
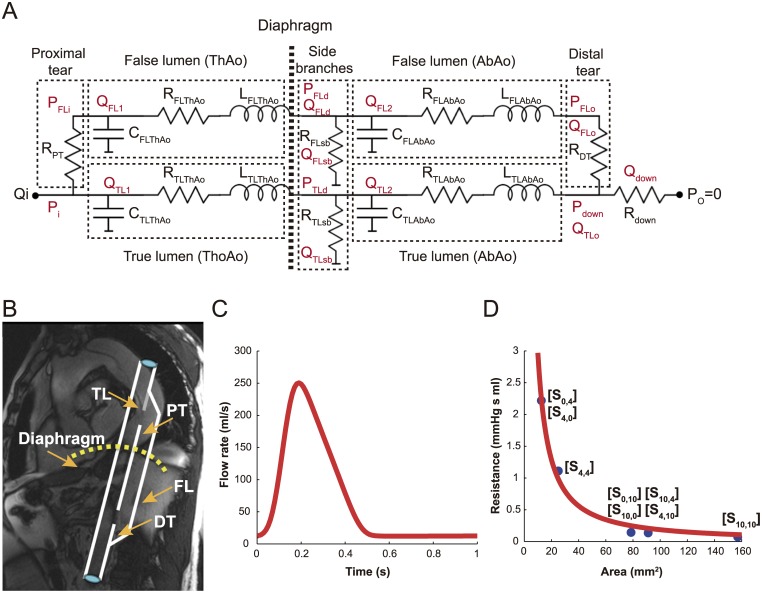
Lumped-parameter computational model of an aortic dissection. (A) Diagram of the lumped-parameter model of an aortic dissection including the presence of abdominal side branches (sb) and the modeling of the thoracic (ThAo) and abdominal (AbAo) aorta; (B) Clinical appearance of an aortic dissection with magnetic resonance imaging and the equivalent dissection geometry proposed; (C) Imposed inflow curve; (D) Value of interluminal resistance plotted as a function of the cumulative tear area, resulting from the calibration of the lumped-parameter model to the experimental in-vitro one [17]. Each data point corresponds to the cumulative value of the tear resistances in the numerical model related to the cumulative tear area in the experimental model for a specific scenario S_P,D_; P, proximal tear diameter; D, distal tear diameter; FL, False lumen; TL, True lumen; PT, Proximal tear; DT, Distal tear.

For our computational study, we chose a typical AD as baseline case. It consisted of two parallel channels (Fig 2B): TL (8.8 mm inner radius; 1.6 mm wall thickness) and FL (15.5 mm inner radius; 0.8 mm thickness). Lumina were communicated by circular large proximal and distal tears, which is a reasonable approximation to what is observed in chronic dissections [14] especially when focusing on temporal variations of intraluminal flow directions along the dissection. The thoracic aorta (ThAo: 0.106 m length) extended from just below the left subclavian artery to the diaphragm and the abdominal aorta (AbAo: 0.239 m length) from the diaphragm to just above the beginning of the iliac arteries (most patients show dissections extending along both ThAo and AbAo). The luminal radii corresponded to the average observed in our population [2]. Wall thicknesses and lengths were taken from literature [3,15,16].

### Computational model

We used an extension of our lumped-parameter model of an AD (Fig 2A) [17]. The model has been validated with in-vitro measurements from experimental models [3] and can provide accurate predictions of intraluminal pressures and flows in ADs.

Briefly, the (electrically equivalent) model is based on flow in a compliant cylindrical vessel and uses a simplification of the Navier-Stokes equations for incompressible fluid. TL and FL were modeled as parallel compartments connected by resistances to mimic a proximal (R_PT_) and distal tear (R_DT_). Each compartment was an individual L-type filter with local resistance to flow (R_compartment_), wall compliance (C_compartment_) and inertance to flow (L_compartment_) [18,19], computed according to the equations below:

$$R_{\text{compartment}}=\frac{8\mu l}{\pi r^{4}}$$ (1)

$$L_{\text{compartment}}=\frac{\rho \iota }{\pi r^{2}}$$ (2)

$$C_{\text{compartment}}=\frac{3\pi r^{3}l}{2Eh}$$ (3)

where *l* and *r* are the compartment length and radius; *μ* and *ρ* represent the fluid dynamic viscosity (0.004 Pa s) and density (1050 kg/m^3^); *E* the wall Young’s modulus; and *h* the wall thickness.

Some modifications included the division of the dissected segment into ThAo and AbAo with corresponding lumina: TL_ThAo_, FL_ThAo_, TL_AbAo_ and FL_AbAo_. This enabled to evaluate flows and pressures at proximal, diaphragm and distal levels. Moreover, to account for the presence of visceral side branches originating from each of the lumina, resistances R_TLsb_ and R_FLsb_ were added at the AbAo to control the amount of blood going to abdominal arteries connected to TL and FL, respectively.

To define the tear resistances as a function of the cumulative area of luminal communication, the calibration of the lumped-parameter model [17] to the experimental models was used [3]. The cumulative area of communication and the corresponding total tear resistance value were computed for the 8 morphologic scenarios studied in Rudenick et al. [17] (Fig 2D). Since there is an inverse relation between cumulative area of communication (mm^2^) and resistance value (mmHg ml s), an inverse power curve was fitted:

$$Resistance=K\;Area^{b}$$ (4)

with best: K = 47.42 and b = -1.20 (R^2^ = 0.99).

For the inlet flow (ThAo at pulmonary artery bifurcation), a slightly simplified version from a typical patient and values from literature [15] were used (Fig 2C), with mean flow rate: 68 ml/s and peak systolic flow rate: 250 ml/s. All simulations were performed imposing the same inflow and assuming a venous zero-pressure at the termini of the visceral side branches and AbAo.

Total peripheral vascular resistance consisted of the sum of the resistances at the abdominal side branches of the lumina (R_TLsb_, R_FLsb_) plus the resistance corresponding to the vascular bed below the AbAo (R_down_). It was estimated by dividing a mean pressure of 100 mmHg by the mean inflow.

The reference case had circular large proximal and distal tears (~10 mm diameter each), since this is most prevalent in clinical practice [20]. Visceral side branches were only originating from the TL (no abdominal side branches arising from the FL). The model was calibrated for this case with E = 1.4 MPa segment stiffness to obtain normal inlet systolic (120 mmHg) and diastolic pressure (80 mmHg). TL visceral side branches’ resistance was adjusted to have 65% of flow towards them [21] (R_TLsb_ = 2.3 mmHg ml s) with the remainder 35% towards the iliac arteries.

### Simulations

First, to show variability and the flow pattern at any place from proximal dissection origin towards distal end, we determined flow profiles at several positions along the course of the aorta. For this (in the absence of visceral side branches), we varied the ThAo/AbAo length in the model, thus changing where flows were measured. Then, for our baseline case (including TL side branches), we determined flow and pressure profiles at proximal, diaphragm and distal levels in TL and FL.

Next, the model was used to predict the effects of varying potential key parameters (related to clinical outcome) on intraluminal flows.

To assess the effect of wall stiffness, we increased wall stiffness up to 100% [22].

In order to evaluate a different amount of visceral side branches connected to the FL, abdominal arteries, with 65% of flow towards them (35% remaining of the total flow at the descending aorta enters the terminal aorta) [21], were distributed between TL and FL.

To assess the effect of cumulative tear size, proximal and distal equally sized tears were simultaneously changed to obtain cumulative tear areas of 25 mm^2^ and 300 mm^2^, equivalent to tears of 5.64 mm and 19.55 mm diameter, respectively. Since, in reality, both the aorta as well as the peripheral vessels, change diameter and thus resistance and compliance (mainly through vasodilatation/constriction) to maintain pressures within physiological limits, we changed tear sizes taking into account this adaptive response. Therefore, we evaluated the effect of tear size by on the one hand, starting from a chronically large tear that was acutely partially occluded (for example, after endovascular treatment) and on the other hand, from a chronically stable small tear that acutely tears and increases its size. In these scenarios, we simulated the homeostatic response by TL vasodilatation or constriction (changing diameter by 20% [23,24]). For the change from large to small tear, we decreased tear size from 300 to 25 mm^2^ with 20% TL dilatation. For the increase from 25 to 300 mm^2^, the model was firstly recalibrated (E = 0.125 MPa, R_PT_ = R_DT_ = 1.9927 mm Hg ml s) to maintain the same inlet pressure and next, with the increase in tear size, the TL diameter was decreased by 20%.

Finally, to assess the effect of tear location, a total tear area of 302.57 mm^2^ (baseline case) was differently distributed between proximal and distal tears.

The effect of each parameter studied was assessed at the proximal, diaphragm and distal levels of the dissected segment through analysis of: a) flow patterns; b) proportion of total flow into the FL, irrespective of direction (%TVF = |TVF_FL_| / (|TVF_TL_| + |TVF_FL_|), with |TVF_TL_| and |TVF_FL_| the cumulative sum of the absolute value of the instantaneous volume flow in TL and FL, respectively); c) percentage of systolic (%RSF_FL_); and d) diastolic FL retrograde flow (%RDF_FL_).

## Results

### Flow profiles in patients

Based on the direction of flow in systole and diastole, we could identify 4 characteristic FL flow patterns in our population where representative cases are depicted in Fig 1: B_A_ = systolic biphasic flow and primarily diastolic antegrade flow in 6 patients (18%); B_R_ = systolic biphasic flow and primarily diastolic retrograde flow in 14 patients (42%); M_A_ = systolic monophasic flow and primarily diastolic antegrade flow in 9 patients (27%); and M_R_ = systolic monophasic flow and primarily diastolic retrograde flow in 2 patients (6%). Two patients (6%) did not show any of these patterns, with retrograde flow during the whole cardiac cycle in one case and no FL flow in the other. The clinical outcome correlation of the proposed FL flow pattern classification is the subject of another study.

### Spatial variability of flow

**Fig 3:**
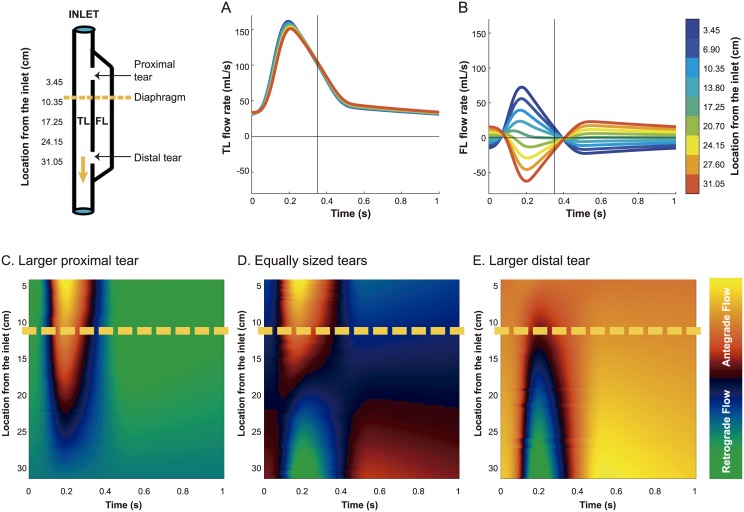
Simulated intraluminal flow patterns along the dissection. Spatial variability of true lumen (TL; A) and false lumen (FL; B,D) flow patterns for the baseline case with 2 large tears and in the absence of any visceral side branches originating from them. The spatial variability of FL flow patterns for the scenarios with a larger proximal (C) and a larger distal (E) tear is also illustrated. Antegrade flows are positive and retrograde flows are negative. Yellow dash lines indicate the diaphragm level.

Fig 3A and 3B shows the variability of flow patterns along both lumina for the baseline case (in the absence of visceral side branches). As can be observed, TL flows (Fig 3A) were very similar and antegrade along the whole segment whereas FL flows (Fig 3B and 3D) showed important changes, with even inversion of the profile, along the dissected segment. At the ThAo, flows were predominantly antegrade in systole and retrograde in diastole, while they inverted when approaching the distal tear, where they were mainly retrograde in systole and antegrade in diastole.

S1A–S1D Fig shows flow and pressure profiles for the baseline case (including TL visceral side branches). Here, the inversion of FL flow profiles at the proximal and distal level can be depicted and, at the diaphragm, an intermediate profile was present. The TL flow had an unchanged profile, with just lower values when distal from the side branches. The pressure profiles did not vary importantly over the length of the aorta, with only a delay and slight increase in TL peak pressure while propagating over the length.

### Wall stiffness

**Fig 4:**
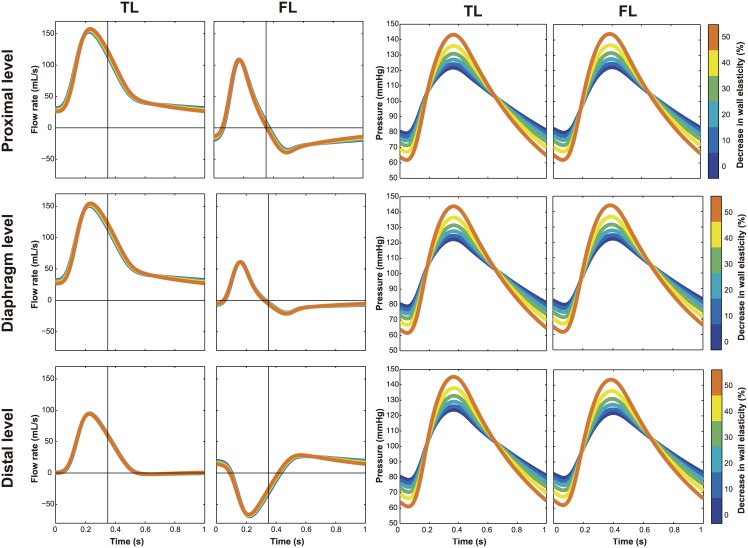
Effect of wall stiffness on intraluminal flows and pressures. Changes in flow patterns (Left) and pressure profiles (Right) with changes in wall stiffness. Antegrade flows are positive and retrograde flows are negative. TL, True lumen; FL, False lumen.

An increase in aortic wall stiffness did not have significant impact on flows at the different levels Fig 4 (Left). Nevertheless, increased stiffness led to a significant increase in pulse pressure in both TL and FL, and as a result, to an augmentation of systolic pressure and decrease in diastolic pressure (Fig 4, Right).

### Incidence of visceral side branches originating from the FL

**Fig 5:**
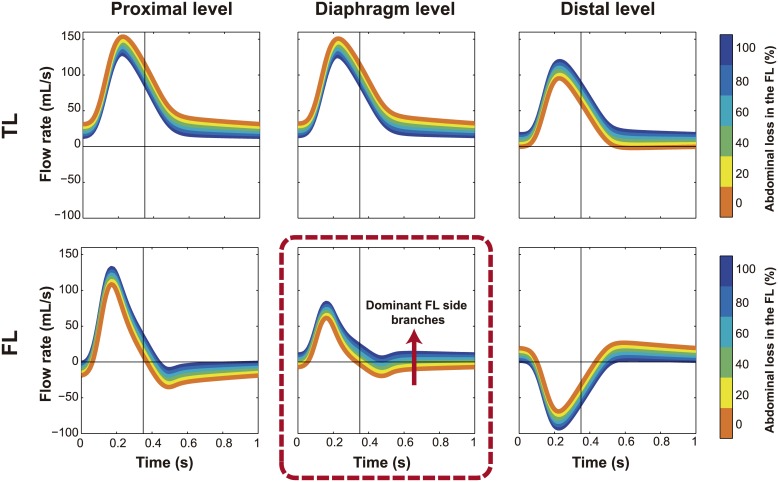
Effect of incidence of visceral arteries arising from the FL on intraluminal flows. Changes in flow patterns with changes in the percentage of abdominal side branches connected to the false lumen (FL). Antegrade flows are positive and retrograde flows are negative. TL, True lumen.

The main effect of an increase in FL side branches was an apparently uniform vertical shift in TL and FL flow patterns at all levels (Fig 5). This affected diastolic FL flow, especially at the diaphragm and distal levels. An increase in FL side branches flattened the diastolic flow, thus decreasing the flow reversal after systole.

Although the presence of visceral side branches at the FL seemed not to significantly affect intraluminal pressures (S2 Fig), there is a trend to lower FL pressures when increasing the incidence of visceral arteries arising from the FL.

### Cumulative tear size

**Fig 6:**
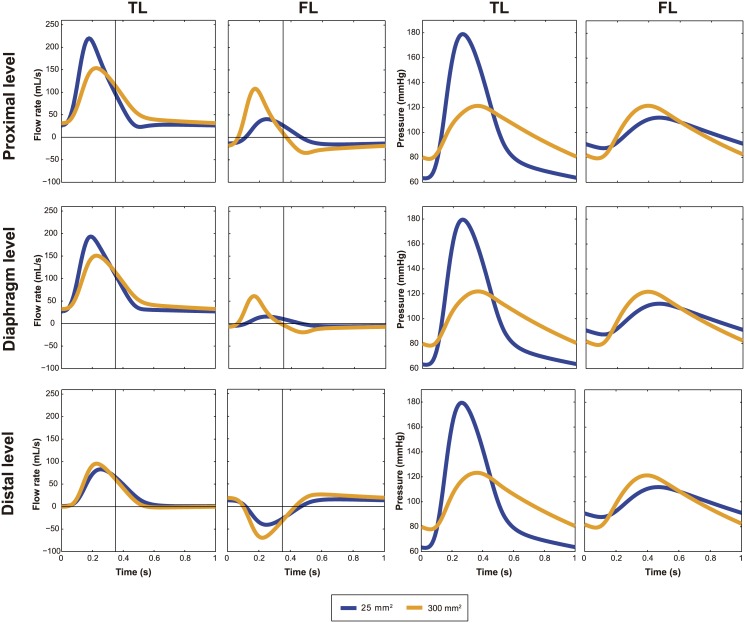
Effect of decrease in cumulative tear area on intraluminal flows and pressures. Variations in flow patterns (Left) and pressure profiles (Right) for a decrease in the cumulative tear area (from 300 to 25 mm^2^) and resultant true lumen (TL) vasodilatation. Antegrade flows are positive and retrograde flows are negative. FL, False lumen.

In both scenarios, flow patterns changed similarly (Fig 6 and S3 Fig, Left). Smaller tear size was associated to a more damped and shifted FL flow with a significant delay in time-to-peak flow compared to TL. Regarding diastolic FL flow, larger tear sizes had more retrograde flow proximally and more antegrade flow distally.

As expected, TL pulse pressure importantly increased when acutely going to a smaller communication (acute hypertension with therapy) and decreased when tear size increased suddenly (acute hypotension with further dissection) (Fig 6 and S3 Fig, Right).

### Spatial distribution of tear size along the dissection

**Fig 7:**
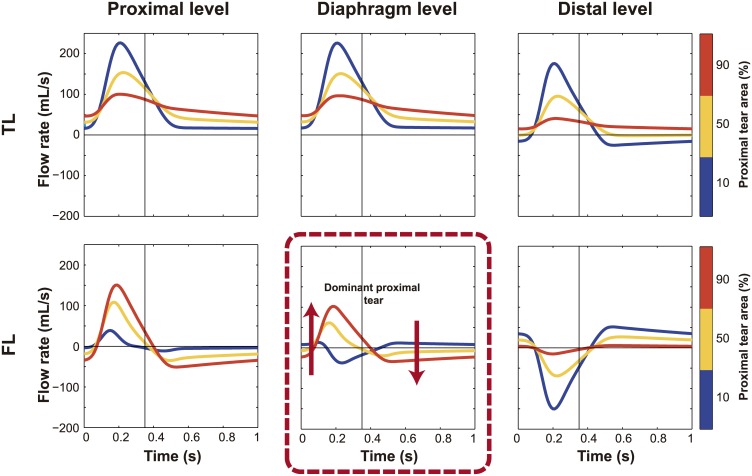
Effect of tear size distribution along the dissection on flows. Changes in intraluminal flow patterns with changes in the distribution of total communicating area between the proximal and distal tears. The color scale represents the percentage of total area distributed at the proximal site. Antegrade flows are positive and retrograde flows are negative. TL, True lumen; FL, False lumen.

Local tear size determined the local proportion of flow going into the FL (Fig 7 and S4 Fig Left). Thus when increasing distal tear area at the expense of proximal, the local flow pattern scaled proportionally, decreasing flow in the proximal site and increasing it at the distal, without affecting the profile shape much. However, this also shifted (from being proximal towards being distal) the place along the dissection where the flow patterns inverted from predominantly antegrade to predominantly retrograde in systole (Fig 3C–3E). The flow inversion point was distal to the diaphragm in presence of a larger proximal tear, and proximal to the diaphragm when the larger tear was at the distal part of the dissection. Thus, when more than 50% of total tear area was located distally, the profile at the (fixed) diaphragm started to show a clear systolic biphasic pattern with an increase in systolic FL reverse flow and mostly antegrade diastolic flows. Instead, systolic FL flows become more monophasic in the presence of a large proximal and small distal tear, with a decrease in systolic reverse flow and an increase in diastolic retrograde flows.

Intraluminal pressures were not significantly affected by redistribution of tear size (S4 Fig).

### Derived parameters

**Fig 8:**
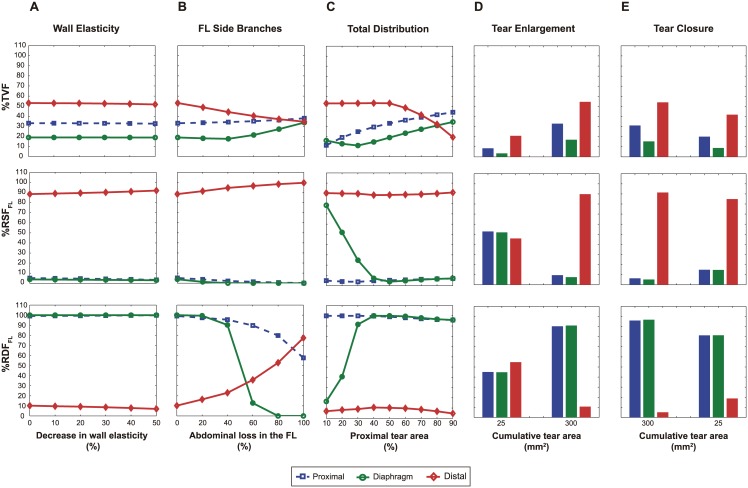
Quantification of flow profiles derived from the parametric study. Percentage of total volume flow into the false lumen (FL) (%TVF), percentage of systolic (%RSF_FL_) and diastolic (%RDF_FL_) FL retrograde flow for changes in (A) wall stiffness of the dissected segment; (B) percentage of FL side branches; (C) distribution of total area between the proximal and distal tear; (D) increase and (E) decrease in cumulative tear area.

Fig 8 shows how quantitative parameters extracted from flow profiles were influenced by the above described changes.

Changes in wall stiffness (Fig 8A) did not change the percentage of total volume flow into the FL (%TVF) nor the percentage of retrograde systolic flow (%RSF_FL_) and retrograde diastolic flow (%RDF_FL_) in the FL.

However, all other changes had a clear influence. FL visceral side branches resulted in a dramatic change in %RDF_FL_, especially at the diaphragm (Fig 8B).

Increasing cumulative tear size increased volume flow into the FL at all levels and changed flow reversal at all sites along the dissected segment (Fig 8D and 8E).

Changing the spatial distribution of the tear size had important and non-linear changes in all parameters, especially at the diaphragm (Fig 8C). Larger proximal tears compared to distal ones resulted in overall more flow into the FL and a reversal of the flow profiles at the diaphragm.

### Comparison with four representative clinical cases

**Fig 9:**
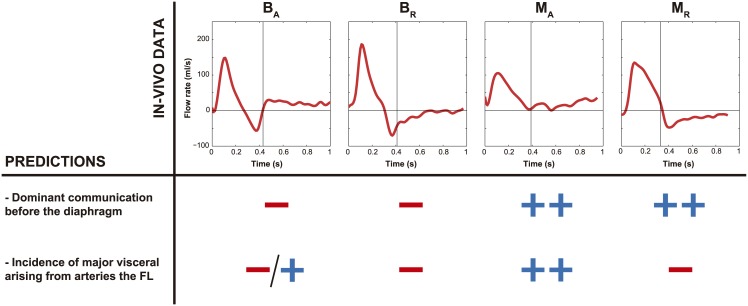
Comparison between in-vivo observations and model predictions. Comparison between the 4 characteristic FL flow patterns (B_A_, B_R_, M_A_, M_R_) identified in the study population and the findings from the parametric study. The different FL flow profiles at the level of the diaphragm can be explained by the percentage of visceral arteries arising from the FL and the location (before/after) of the dominant interluminal communication respect to the place of measurement. Signs illustrate the level of incidence of each property in each FL flow pattern determination: **(-)** absent property; **(++)** existent property; **(+/-)** possible existent property. Antegrade flows are positive and retrograde flows are negative.

The four characteristic instantaneous FL flow profiles at diaphragm level in the total population could be explained by the simulations (Fig 9). From Figs 5–7, S3 and S4 Figs Left, most of the variability in FL flow profiles might be attributed to changes in the spatial distribution of tear sizes and the presence or absence of visceral side branches at the FL.

Table 1 shows the baseline characteristics of 4 randomly selected patients, each representing one of the 4 categories. When comparing patients with systolic monophasic and biphasic FL flow patterns, patients with a systolic monophasic flow profile at the diaphragm had large proximal communications between the lumina and small distal tears while patients with a systolic biphasic pattern showed small proximal tears and larger distal tears (as predicted from Fig 7). In all cases, there was no restricted communication between the lumina, which would have been evidenced from later FL time-to-peak flow compared to the TL (as predicted from Fig 6 Left and S3 Fig Left). When comparing patients with the same systolic flow pattern, the patients with diastolic retrograde patterns did not show major visceral side branches in the FL while the patients with diastolic antegrade patterns did have major visceral side branches in the FL (as predicted from Fig 5). However, for the scenario B_A_, the presence of diastolic antegrade flow in the FL is mostly determined by the presence of an important communication distal to the diaphragm and to a lesser degree by the communication of the FL with visceral side branches. Therefore, for this scenario, it is important to emphasize that the presence of visceral side branches arising from the FL is a possible but not necessary property.

**Table 1:** Baseline characteristics and quantitative flow assessment at the level of the diaphragm for 4 representative cases of the FL flow pattern subgroups.

Representative Patients—Flow Patterns				
	1—B_A_	2—B_R_	3—M_A_	4—M_R_
**Age**	41	27	70	37
**Stanford Type**	Surgically-treated type A	Surgically-treated type A	Surgically-treated type A	Surgically-treated type A
**Extent**	From proximal ThAo to the left iliac artery	From proximal ThAo to the right iliac artery	From proximal ThAo to the end of the AbAo	From proximal ThAo to the end of the AbAo
**Time between Acute Event and MRI (days**)	107	253	223	164
**|TVF**_**FL**_**| (mL/cycle)**	3534	3460	3137	3851
**%TVF**	39.66	44.35	46.38	45.46
**%RSF**_**FL**_	9.31	4.47	0	1.48
**%RDF**_**FL**_	21.28	98.87	0	100
**TTP**_**TL**_ **Flow (s)**	0.18	0.20	0.23	0.22
**TTP**_**FL**_ **Flow (s)**	0.11	0.11	0.11	0.11
**Communication between the Lumina**	Absence of tears of significant size at the ThAo. Presence of tears at the renal and infra-renal levels.	Multiple small tears at the aortic arch, ThAo and AbAo. Not significant large proximal tears.	Main proximal tear with multiple small tears at the ThAo. Absence of significant tears at the AbAo.	Large proximal tear of 3.2 cm^2^ area. Absence of additional significant tears at the ThAo and AbAo.
**Incidence of Visceral Side Branches in the FL**	Left-renal artery and celiac trunk.	Right-renal artery.	Left-renal artery and inferior mesenteric artery.	Right-renal artery.

TL, true lumen; FL, false lumen; TVF, total volume flow; %TVF, proportion of total flow that goes into the FL; %RSF, proportion of FL retrograde systolic flow; %RDF, proportion of FL retrograde diastolic flow; TTP, time-to-peak flow; ThAo, thoracic aorta; AbAo, abdominal aorta.

Therefore, a clear correspondence exists between the results of the simulations and the clinical characteristics of the 4 selected cases. This illustrates the capability of the lumped-parameter computational model to provide further insight into FL flow profiles in ADs.

## Discussion

The current study demonstrates that within a typical population in descending ADs, there are predominantly 4 characteristic FL flow patterns when looking at the direction of flow in systole and diastole at diaphragm level. Moreover, using a lumped-parameter model, we demonstrated that the abdominal arteries communicating with the FL and the spatial distribution of the size of interluminal communications are important determinants of these patterns.

Therefore, from FL flow patterns might be possible to infer morphological information on key determinants of aortic expansion in ADs. Moreover, local flow changes during the cardiac cycle are extremely dependent on the position along the dissection, with a profile inversion in between tears.

Progressive dilatation and potential subsequent rupture are major long-term concerns in chronic ADs. Besides the influence of increased pressures, aneurysmal growth of the aorta has been linked to the amount of communication between the lumina and to FL patency [2,5]. Therefore, imaging-based assessment of flow in individual patients, together with a better understanding of determinant mechanisms, may be useful for predicting enlargement and future complications. It is important to emphasize that our main goal was to elucidate on the origin of the most prevalent hemodynamic patterns observed in chronic ADs and not the correlation between these patterns with long-term aortic enlargement and clinical complications, which is the subject of another study.

For the study, we used a lumped-parameter computational model able to assess realistic local flows in a diverse range of geometric and biophysical variations. To our knowledge, it is also the only validated lumped-parameter computational model of an AD [17] that can capture a wide variety of influencing factors, including e.g. presence of visceral side branches. Compared with three dimensional fluid simulations, lumped-parameter models represent a complex vascular structure by means of a single tube with properties representative of the whole system, so that they lack the spatial dimension and only bring average pressure and flow information of a whole vascular segment instead of detailed local hemodynamic information. However, different from CFD (with rigid wall approach), lumped-parameter models include the notion of wall elasticity, which is a key factor in the modeling of ADs and the resulting intraluminal hemodynamics [17, 25]. Including elastic walls in 3D simulations requires fluid structure interaction and dramatically increases model complexity and computational cost. Additionally, the utility and accuracy of a lumped-parameter model do not depend on the spatial information of the vasculature as in CFD, which makes it easier to implement, makes it computationally much less expensive, and thus more versatile to perform an extensive parametric study as compared to CFD. Therefore, a lumped-parameter model is suitable for the aim of the current study, which consists of determining the relevant parameters affecting/determining the hemodynamics in ADs and learning about the average rather than local flow characteristics of the system, without the need of going into a very detailed and expensive model.

The characteristic FL flow patterns observed in the MRI measurements in patients could be reproduced by our model and the predicted factors determining the different temporal profiles during the cardiac cycle were indeed present, with characteristic changes in the direction of the FL flow that could be partly attributed to changes in presence of FL side branches and position/size of interluminal communications.

Our simulated flows (Table 2) had a number of similarities with MRI measurements, in concordance with our clinical observations (Table 1) and François et al. [12] Total TL flow volume was generally larger than FL and TL flows were mostly antegrade over the dissection, with time-to-peak flow rate delayed compared to the FL. However, FL flows were retrograde either depending on the position along the length of the dissection or the time point in the cycle. Reversed FL flow occurred in late systole or diastole in ThAo, as also observed by Chang et al. [26] and Strotzer et al. [10]. Only close to the distal part of the dissected segment was FL systolic flow always predominantly retrograde.

**Table 2:** Quantitative flow assessment at the proximal, diaphragm and distal levels of the dissected segment for the baseline case of a chronic dissection.

	Proximal Level		Diaphragm Level		Distal Level	
	TL	FL	TL	FL	TL	FL
Total Flow (mL/cycle)	6742.2	3295.5	6740.6	1532.7	2435.5	2759.0
Peak Flow Rate (mL/s)	154.32	106.39	151.69	59.56	96.44	70.35
Time to Peak Flow (s)	0.22	0.17	0.23	0.16	0.23	0.22

TL, True lumen; FL, False lumen.

The impact of tear size/location in determining hemodynamics has already been the subject of experimental and clinical studies [2,3,27,28]. However, a more systematic and thorough analysis of tears and their correlation with flow phenomena was still lacking. We found that a large communication between the lumina increased the proportion of FL flow volume and affected systolic and diastolic FL flow reversal at the proximal and distal sites. Moreover, a decrease in total communication area resulted in a more damped FL flow curve and delayed time-to-peak flow compared to TL, which is due to the damping effect of the tears, inhibiting fast changes in flow. Additionally, in agreement with our previous studies [3], cumulative tear size significantly determined pressures and the exact anatomic configuration where they were located (large proximal or distal tear) played a secondary role. Although the spatial location of the size of tears did not influence pressures, it did importantly determine flow profiles, mostly around the center of the dissection (thus at the diaphragm level, which is currently a standard position to acquire MRI flows). When the proximal communication was sufficiently smaller than the distal one, flows at the diaphragm showed a characteristic systolic biphasic pattern with an increase in systolic retrograde flow and mainly diastolic antegrade flow. When the proximal tear turned larger than the distal, systolic FL flow turned more monophasic with a decrease in systolic retrograde FL flow together with predominantly diastolic retrograde flow. As expected, local tear size was directly related to local amount of flow.

Since many AD patients have visceral side branches (including important ones such as the celiac trunk) originating from the FL, and this was reported to be significant related to aortic enlargement [5,29], we assessed their influence on flow. Our results showed that the incidence of FL side branches increased the amount of FL flow volume and affected FL flow direction, so that FL flows became more unidirectional (mostly antegrade or retrograde depending on the level of the dissection), primarily affecting the percentage of retrograde diastolic FL flow. This is consistent with Inoue et al. [5] (taking into account that their measurements were from the central part of the FL) where patients with mostly antegrade and retrograde patterns showed a higher incidence of abdominal arteries originating from the FL than those with bidirectional flows, and patients with antegrade patterns had more FL flow. On the other hand, visceral side branches connected to the FL did not significantly affect pressures (with a slight decrease in FL pressures when increase), which suggests that they might be associated to aortic expansion because of their effects on flows rather than pressures, considering that the total flow and complexity of FL flow have been previously related to dilatation [5,9,11].

Changes in wall stiffness have been also reported as a potential factor for pressures and wall shear stress (WSS) [13,30] and might play an important role in hemodynamics and dilatation in ADs. This parameter is altered in most patients, since the majority has abnormal aortic elastic properties from natural aging or factors, such as hypertension, genetic disorders (e.g. Marfan) and atherosclerosis. In our model, the effects of variations in stiffness were not influencing local flow patterns but were very important for determining pressure, with a significant increase of systolic and pulse pressure in stiffer arteries. The pulse pressure was increased by 61% and 92%, with 40% and 50% stiffness increase respectively, consistent with the findings of Reymond et al. [31] and Johnson et al. [32].

### Implications

Our results demonstrate the high complexity and variation in FL flow compared to TL flow and its relation to geometrical and biophysical properties of the dissected aorta, implying that measurements of FL flows could help in assessment and prognosis in chronic AD.

Most information seems to be in the FL flow profile, but assessment of the TL is also important to compute the proportion of volume towards each, an index already suggested to predict aortic enlargement [5].

Although pressures showed no large changes depending on where they were assessed, FL flow was importantly influenced by the point along the dissection where it was measured. This implies that measuring at only one level (e.g. diaphragm or dissection center) will not enable understanding the whole complex flow variation, especially since there is an inversion of the flow along the dissection length in most patients. A comprehensive imaging study to assess flow should therefore measure them at several places along the dissection.

Nevertheless, our study suggests that FL flow patterns at the diaphragm level are able to depict changes in the distribution of interluminal communications along the dissected segment and the presence of FL side branches, both important determinants of aortic expansion and modifiable features in ADs and therefore, with high clinical relevance. Thus, a measurement of the FL patterns at the diaphragm could give a first general insight into the individual patient and help clinicians in inferring the potential factors responsible for these patterns while suggesting a deeper analysis if needed. For pressures, only wall stiffness influenced them and the effects could not be noticed from the mere analysis of flow. However, flows were determined by the presence of FL visceral side branches or the spatial distribution of total tear area. Therefore, since all these mechanisms coexist in a patient, each needs to be carefully addressed when assessing hemodynamics for prognosis in ADs.

The model also provides insights into less familiar features that could be of potential importance in the clinical assessment, management and response of patients, such as the wall stiffness and thus mobility of the dissection flap; or the preservation of visceral branches arising from the FL in order to make FL flows more unidirectional (decreasing oscillatory wall shear stress and so adverse endothelial remodeling and aortic dilatation) and slightly decrease FL pressures.

Eventually, the characterization of FL flow patterns with underlying anatomical and biomechanical features of the AD could be used in practice combined with the knowledge resulting from the clinical correlation between the flow patterns identified in this study and the long-term aortic enlargement/complications in patients.

### Study limitations

The inflow curve and the dissection model have been constructed based on values corresponding to an average patient in chronic type B AD, so that the model predictions would reflect the characteristics of an average population rather than a specific patient’s. Therefore, given the differences between a specific patient and the computational model, the study is aimed to gather further insight into overall hemodynamics in AD more than the direct translation of the findings to the clinical practice.

The model used is a simplification of anatomical reality. ADs can be very complex with tortuosity; irregularities of lumina diameter along the dissection; helicoidal intimal flap; complex communication between lumina where iliac arteries can be originating from the FL; partial FL thrombosis; flap mobility, among others. Additionally, a lumped-parameter model does not account for wave propagation phenomena. These aspects could be important in exact local flow (and pressure or WSS) determination. Nevertheless, the simplifications are justified for the current purpose of characterizing overall flow profiles within a cross-section and dependency on changes in different key parameters.

The same synthetic inflow curve has been imposed in all the cases to study the isolated influence of each parameter in the absence of hemodynamic alterations. It implies that the selected wave will lead to pressure and flow waves resulting of the particular interaction between the inflow curve imposed and the current vasculature. Despite not being in the scope of the study and so not reported, we have also performed the parametric studies imposing different inflow curves (including patient-specific ones) obtaining comparable flow and pressure properties for all the cases, without changing the conclusions of the study. Moreover, the assumption of a constant inflow curve, and so constant cardiac output, implies an increased workload on the heart. However, most of patients with chronic type B ADs undergo medical control of blood pressure and heart rate within the normal average values [33] so the cardiac output remains stable without important variability among patients.

The study of spatial distribution of tear size has been performed using only 2 tears and without varying the distance between them. However, pressures are determined by the accumulative area of the communicating tears rather than by the size of/ distance between the local communicating tears [3,28,34], and the FL flow profile at the place of measurement will be the result of the competition between the total communicating areas located proximal and distal to that place, independent on the number of tears and their distribution in each case.

Terminal vascular beds have been modeled as pure resistances instead of three element Windkessel models, since the inclusion of a compliant bed did not change the conclusions of the study in a limited sensitivity study performed. In addition, each lumen was modeled with a Poiseuille resistor, simplification justified when studying global flow phenomena in the setting of pulsatile flows in large arteries [35,36].

A further validation of the lumped-parameter model predictions with our large patient population, to carefully assess the correlation between FL flow patterns by MRI and the modifiable anatomical features predicted by the model, as well as the integration of this information with their prognostic value, are not reported here.

## Conclusions

Our study gives novel insights into potential causes of both spatial and temporal variation in FL flow patterns in patients with chronic ADs and thus provides a new basis to correlate these changes observed in MRI flow measurements to their underlying causes. It is also the first study to favorably compare computational predictions with clinical cases. Intraluminal flow patterns in chronic ADs, especially their temporal variation with directional changes, depend on position of assessment as well as size and spatial distribution of tears and visceral side branches. Therefore, a standardized imaging protocol measuring local flow patterns can give useful insights into individual patients and might aid with their clinical management and assessment of prognosis.

## Supporting Information

S1 Fig Baseline intraluminal flow and pressure profiles. Flow (A-B) and pressure profiles (C-D) in the true lumen (TL) and the false lumen (FL) assessed at the proximal, diaphragm and distal levels, for the baseline case of a chronic aortic dissection (including TL visceral side branches). Antegrade flows are positive and retrograde flows are negative.
(EPS)

S2 Fig Effect of incidence of visceral arteries arising from the FL on intraluminal pressures. Changes in true lumen (TL) and false lumen (FL) pressure profiles with changes in the percentage of abdominal side branches connected to the FL.
(EPS)

S3 Fig Effect of increase in cumulative tear area on intraluminal flows and pressures. Variations in flow patterns (Left) and pressure profiles (Right) for an increase in the cumulative tear area (from 25 to 300 mm2) and resultant true lumen (TL) vasoconstriction. Antegrade flows are positive and retrograde flows are negative. FL, False lumen.
(EPS)

S4 Fig Effect of tear size distribution along the dissection on flows and pressures. Changes in true lumen (TL) and false lumen (FL) flow patterns (Left) and pressure profiles (Right) with changes in the distribution of total communicating area between the proximal and distal tear. The color scale represents the percentage of total area distributed at the proximal site.
(EPS)
